# Investing in women, girls, and other matters

**DOI:** 10.4103/0972-2327.56310

**Published:** 2009

**Authors:** Sanjeev V. Thomas

**Affiliations:** Editor, Annals of Indian Academy of Neurology and Professor of Neurology, Department of Neurology, SCTIMST, Trivandrum, India. E-mail: editor@annalsofian.org

Investing in women and girls is the theme for the World Population Day (11^th^ July) this year. The world population now stands close to 6.8 billion and it is projected double in the next 40–50 years. The consequences of population explosion on this planet and possibly the universe itself are inconceivable.

The health status of women and girls in India deserves much more attention than it currently receives. There are several neurological disorders that affect females more than males. In India, women fail to avail of modern neurological services. In this current phase of economic recession, the United Nations has rightly focused attention on women and girls in order to contain population growth and improve quality of life. Mr. Ban Ki Moon, the Secretary General of United Nations, has called upon the decision makers ‘to protect women's ability to earn incomes, keep their daughters in school, and obtain reproductive health information and services, including voluntary family planning.’ He has appealed for advancement of the rights of women and girls and their empowerment as highly productive members of society capable of contributing to economic recovery and growth.[1]

We will be observing 21^st^ September as World Alzheimer's Day. The theme for this year – ‘Diagnosing Dementia: See It Sooner’ – is very pertinent to our profession. In this issue of the journal we have published an important original article on the incidence of dementia in Kashmiris. Incidence studies are, in general, sparse from our country. The authors of this paper deserve special commendation for carrying out their epidemiological study in this special subgroup of migrant Kashmiris. We need to standardize our tools to screen Indian populations for Alzheimer's disease and other related disorders. Several population-based epidemiological studies are urgently required before we can develop strategies to make early diagnosis of Alzheimer's disease and other dementias. The role of environmental factors, malnutrition, and other social factors specific to the Indian subcontinent in the pathogenesis of this group of disorders deserve systematic evaluation.

This issue of the journal carries several important original articles and review articles that should make interesting reading. I take this opportunity to call upon the readers to interact more actively by sending your comments and experience in the form of letters to the editor.
